# Successful Repair of a Strasberg E4 Bile Duct Injury With Double-Barreled Dual Hepaticojejunostomy: A Case Report

**DOI:** 10.7759/cureus.99147

**Published:** 2025-12-13

**Authors:** Monther Nassar, Vincent Smith, Dalun Tang, Jianlin Tang

**Affiliations:** 1 Surgery, University of Toledo, College of Medicine and Life Sciences, Toledo, USA

**Keywords:** bile duct injury, double barrel dual hepaticojejunostomy, laparoscopic cholecystectomy, roux-en-y, strasberg e4

## Abstract

Bile duct injury (BDI) is a catastrophic complication of cholecystectomy that can profoundly affect a patient’s quality of life. While hepaticojejunostomy is the standard surgery for major BDI repair, reports of double-barreled dual hepaticojejunostomy following Strasberg E4 injury are limited. The complication rate of the repair of BDI above the bifurcation is very high, and very dismal outcomes are reported. We present a case of a 79-year-old male patient with multiple comorbidities who underwent laparoscopic cholecystectomy for acute gangrenous cholecystitis. He was transferred to our hospital on postoperative day (POD) 3 due to bile leak and sepsis. Endoscopic retrograde cholangiopancreatography* *(ERCP) showed a large number of clips on the hilum of the liver and distal common bile duct obstruction. Exploratory laparotomy identified a missing common bile duct and hepatic duct above the confluence, with less than 5 mm extrahepatic left and right hepatic duct stumps left, which were occluded by multiple clips. Also, there was a sign of cauterization at the end of the stumps. Patient’s condition did not permit safe intrahepatic dissection. Following the removal of clips and debridement of the hepatic duct stumps, a Roux-en-Y double-barreled dual hepaticojejunostomy was performed over stents. Postoperatively, the patient had a transient bile leak, tolerated diet, and was discharged to a skilled nursing facility on POD 12 and remained asymptomatic six months postoperatively. In this case, Strasberg E4 BDI resulted in separated left and right hepatic duct stumps, which require a double-barreled dual hepaticojejunostomy to address multiple hepatic duct stumps. Multiple anastomoses, smaller stumps, and the narrow space inside the liver hilum significantly increased the difficulty of the repair. Due to the rarity of double-barreled dual hepaticojejunostomy, further studies are needed to assess its feasibility, mortality, and morbidity.

## Introduction

Bile duct injury (BDI) is one of the most catastrophic complications after cholecystectomy. It has a disastrous negative impact on patient life expectancy and health-related quality of life, and it increases health care costs, resulting in a high rate of litigation. In open cholecystectomy, the incidence of BDI is 0.1-0.2%. The advent and refinement of laparoscopic cholecystectomy have significantly reduced recovery time and morbidity after cholecystectomy. However, the incidence of serious BDI is higher, ranging from 0.15% to 0.36% [1].

Several classification systems have been developed to guide the treatment of BDI. The original and widely accepted Bismuth-Corlette classification was created during the era of open surgery to categorize hilar cholangiocarcinoma; it was also used to categorize extrahepatic BDI [2,3]. It aimed to guide the selection of appropriate surgical reconstruction techniques and has shown good correlation with outcomes [4]. However, the Bismuth classification does not encompass the full spectrum of bile duct injuries, particularly those encountered in laparoscopic surgery [2]. It was later modified by the Strasberg classifications in 1995 [5]. The Strasberg classification focuses on extrahepatic bile duct injuries and bile leaks. Strasberg E4 injury is the transection or stricture above the common hepatic duct confluence. It is the most severe BDI.

The best timing of BDI repair is controversial. Some literature found that the timing of repair has no significant impact on the outcome [6,7]. Another author found that BDI repair had the worst outcome between eight days and two weeks after the initial BDI [8]. However, there is literature that confirms that timely diagnosis and repair of BDI play a crucial role in achieving an optimal outcome, provided control of sepsis is also achieved simultaneously [9]. Intraoperative detection often leads to better outcomes [10]. Whereas postoperative diagnosis of missed BDI relies on clinical signs (e.g., jaundice, bile leak, sepsis), imaging modalities such as endoscopic retrograde cholangiopancreatography (ERCP) and surgical exploration are also utilized [2,10,11].

While nonoperative interventional radiology or endoscopic biliary drainage procedures are often used to manage sepsis or bile leak initially, definitive repair is still accomplished with Roux-en-Y hepaticojejunostomy, especially in cases with significant tissue loss [11]. Although hepaticojejunostomy is a standard surgery for major BDI repair [12], the complication rate of repairing BDI above the hepatic duct confluence was more than 50% [13]. Kapoor et al. reported the dismal result of fixing the Strasberg E4 injury with hepaticojejunostomy, in which nine of 10 patients developed cholangitis after repair. Five of these underwent hepatectomy, one underwent liver transplantation, and another three underwent radiology intervention [14].

We report the successful primary repair of a Strasberg E4 BDI, combined with clip and thermal injury to the hepatic duct stumps, using a double-barreled dual Roux-en-Y hepaticojejunostomy, an approach rarely reported in the literature. This case will serve as part of the data accumulation for further investigation to improve outcomes in complicated BDI. 

The work has been reported in line with the SCARE (Surgical CAse REport) 2025 criteria [15].

## Case presentation

Preoperative

**Figure 1:**
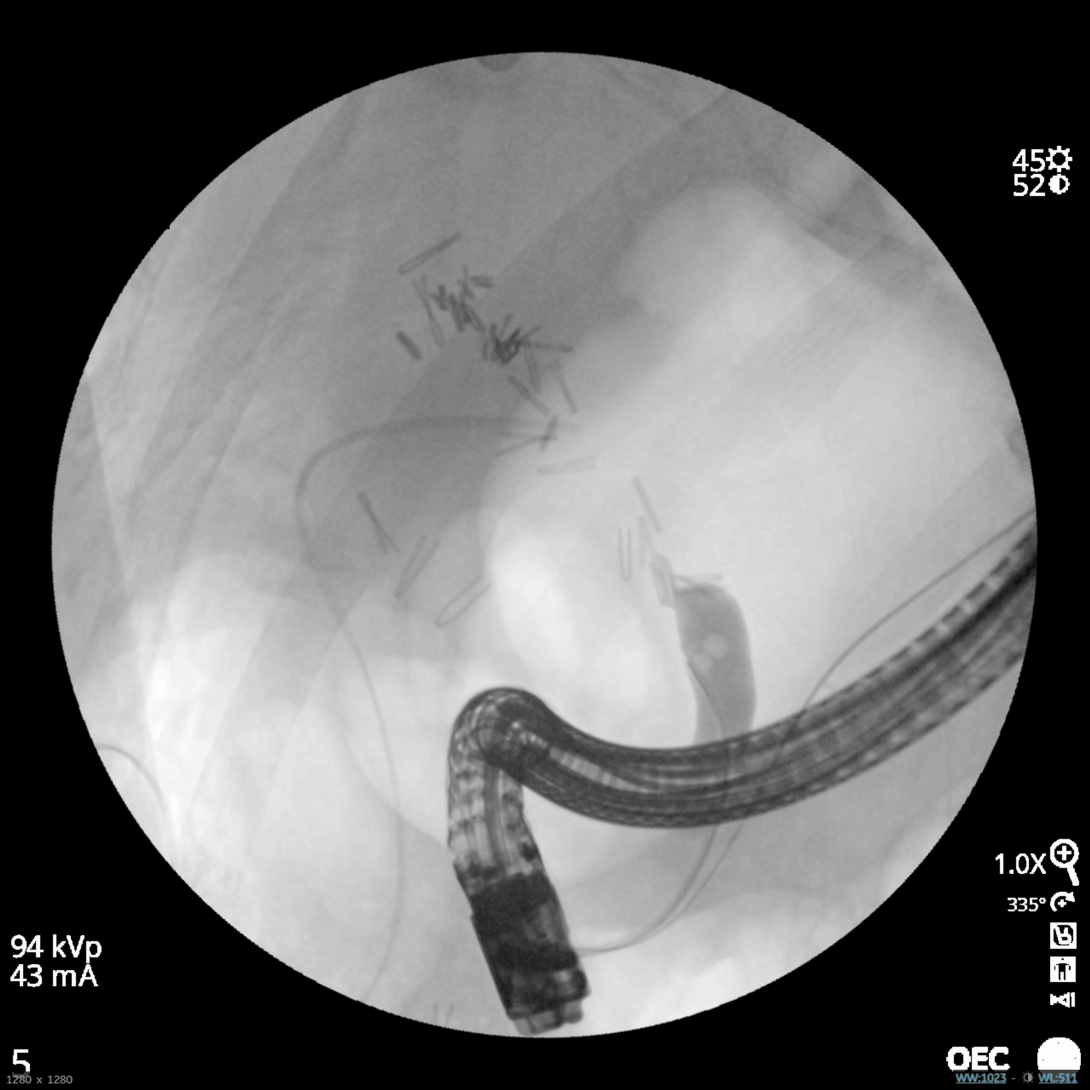
ERCP demonstrating filling defect of CBD and large number of surgical clips on hilum of the liver and distal common bile duct obstruction, unable to advance the wire ERCP: endoscopic retrograde cholangiopancreatography; CBD: common bile duct

A 79-year-old male patient was referred to our tertiary hospital in April 2025. Past medical history included type 2 diabetes mellitus, atrial fibrillation, myocardial infarction, dementia, malnutrition, and benign prostatic hyperplasia. He had undergone a laparoscopic cholecystectomy three days prior, for an acute gangrenous cholecystectomy. The surgical report was notable for difficult dissection, significant blood loss, top-down dissection, and the use of ultrasonic shear as an energy instrument. An intra-abdominal drain was left in place with subsequent biliary output. ERCP revealed numerous clips along the hilum of the liver and distal common bile duct obstruction (Figure 1).

General surgery was consulted for possible BDI. Acute comorbidities include sepsis with encephalopathy, leukocytosis, and atrial fibrillation with rapid ventricular response. Total bilirubin level was within normal.

Intraoperative

**Figure 2:**
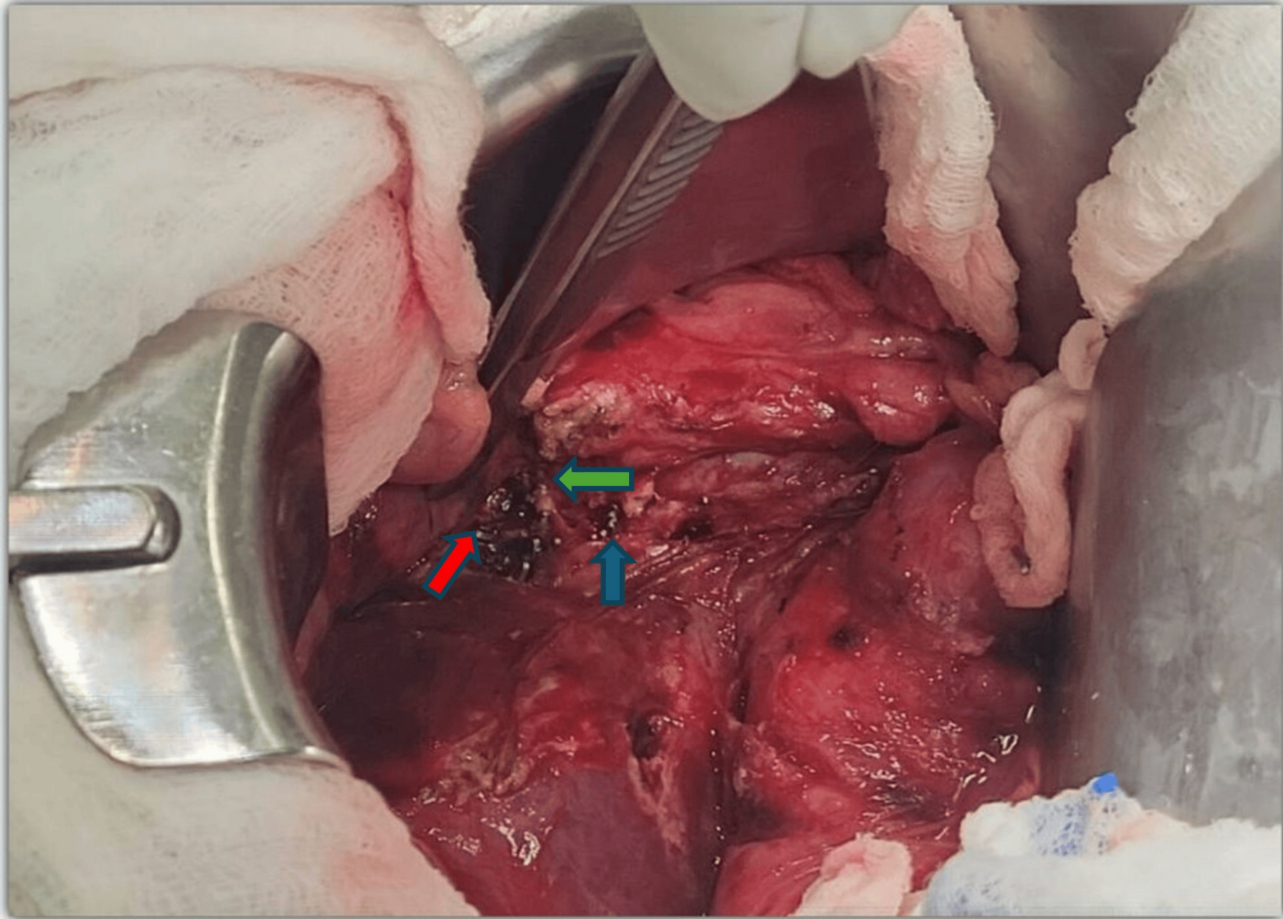
Intraoperative image showing missing common bile duct and hepatic duct above hepatic duct confluence, clips and thermal injury to left and right hepatic duct stumps Green arrow: left hepatic duct stump; Red arrow: right hepatic duct stump; Blue arrow: clips on portal vein

The patient was admitted to the surgical ICU; IV antibiotic therapy and fluid resuscitation were performed before the surgery. Operative exploration was performed via a chevron incision. Exploration of the gallbladder fossa and porta hepatis revealed an absent common bile duct and common hepatic duct above the confluence (Figure 2). More than 20 metal clips are present along the porta hepatis. The right and left hepatic ducts were transected at the liver hilum with thermal injury and surgical clips. This is evident in the Strasberg E4 injury.

The surgical clips were sequentially removed, and hemostasis along the portal vein was achieved using 6-0 Prolene sutures. The right and left hepatic duct stumps were cannulated with 6 cm long 8 Fr red rubber catheters as a stent. The bile duct ends were sharply debrided due to thermal injury. Debridement was challenging with a minimal hepatic duct length of less than 5 mm. Hemodynamic instability did not permit a longer operative time or trauma of intrahepatic dissection. Due to the distance between the right and left hepatic ducts (more than 1 cm) and the level of disruption, a decision was made to make two separate anastomoses. A Roux limb was created in retro-colic fashion. Both anastomoses were created using simple interrupted 4-0 PDS (polydioxanone) sutures. A red rubber catheter was placed within each anastomosis to function as a stent. An intra-abdominal drain was placed. The smaller size of the hepatic duct stumps and the very narrow space inside the liver hilum significantly increased the difficulty of the anastomosis.

Postoperative

**Figure 3:**
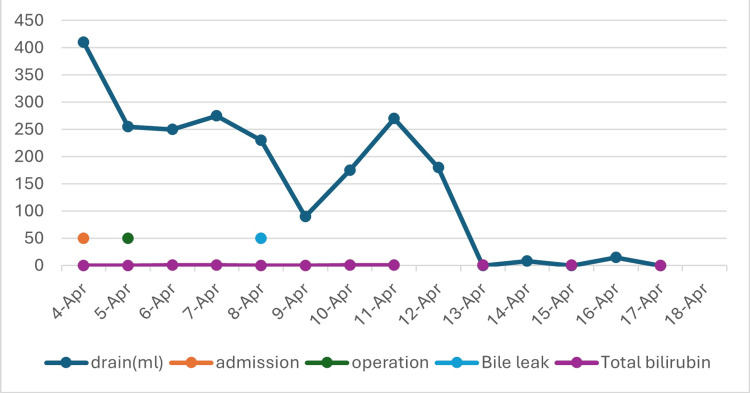
Perioperative peritoneal drain output and serum bilirubin level

The patient was monitored postoperatively in intensive care settings. Parenteral nutrition was started for severe protein malnutrition. Bile leak noted on postoperative day (POD) 3, which resolved without further intervention five days later. Diet was advanced on POD 7 after resolution of ileus. The patient was discharged to a skilled nursing facility on POD 12. During the two-week follow-up, the patient continued to tolerate a regular diet, staples and drains were removed without further interventions, and bilirubin was within normal limits (Figure 3). At the six-month postoperative telephonic follow-up, the patient remained asymptomatic, without jaundice, fever, or weight loss.

## Discussion

Strasberg E4 injury is the most severe BDI, which includes loss of hepatic duct confluence, frequently loss of most of the extrahepatic bile duct [5]. Reports on the incidence, presentation, treatment options, and outcomes of Strasberg E4 injuries are limited. Mercedo et el. reported 53 cases of Strasberg E4 injury out of 603 cases of BDI [13]. The incidence of Strasberg E4 injury is low, around 10% of all iatrogenic BDI from laparoscopic cholecystectomy [13,16]. However, there were three cases of Strasberg E4 injuries referred to our institution in the last two years.

The repair of the Strasberg E4 injury is very challenging. The presence of small, multiple hepatic duct stumps, the narrow confines of the liver hilum, and jejunum edema pose significant challenges to the repair. According to Mercedo's report, there are three types of reconstruction techniques: (i) Neoconfluence reconstruction: This reconstruction includes reunited left and right hepatic ducts, then opening the anterior wall of the hepatic duct for hepaticojejunostomy. This reconstruction technique delivered the best outcome; (ii) Portojejunostomy: This technique was employed in cases where hepatic duct stumps can not be fully identified. The anastomosis is between the portal plate and jejunum, with less than 50% of the circumference reconstructed using a high-quality ductal-to-jejunal mucosa anastomosis. The outcome of this technique was poor, and the postoperative complication rate was 57.6% with 53.8% patients developing cholangitis. Postoperative cirrhosis rate was 61.5% and 50% patients were referred for liver transplantation; and (iii) Double-barreled dual hepaticojejunostomy: This technique was employed in the case of left and right hepatic duct stumps that could not be reunited; two separate hepaticojejunostomies were performed. There were only six such cases in Mercedo's report. The major postoperative complication was cholangitis [13]. Kapoor et al. also reported a dismal outcome for Strasberg E4 BDI repair, with a 90% rate of postoperative cholangitis [14].

Our patient's injury included loss of confluence of the hepatic duct; his right and left hepatic duct stumps are more than 1 cm apart. We decided to perform a double-barreled dual hepaticojejunostomy over stents to address multiple hepatic stumps. The narrow space at the liver hilum barely accommodated the jejunal limb; the anastomosis may have been under some tension, resulting in a transient postoperative bile leak. Fortunately, the bile leak resolved spontaneously, and the patient recovered fully. The patient remained asymptomatic six months postoperatively. Our result is significant in light of the dismal outcomes of previous Strasberg E4 BDI repair [13,14].

Several factors have contributed to our good outcome. Early repair is essential; we can clearly identify the right and left hepatic duct stumps and perform a high-quality anastomosis. We chose a double-barreled dual hepatocystojejunostomy to achieve an optimized anastomosis and successfully address the multiple hepatic duct stumps. The most important factor is the high-quality anastomosis over a stent, which directly translates to a superb outcome. Due to the low incidence of Strasberg E4 injuries and the rarity of dual hepaticojejunostomy, further studies and data collection are needed to assess the feasibility of this procedure and its associated mortality and morbidity.

## Conclusions

Our experience supports primary double-barreled dual hepaticojejunostomy as a feasible option for managing Strasberg E4 BDIs after adequate resuscitation and antibiotic therapy, even in elderly patients with comorbidities. This case demonstrates that, with proper expertise, complex BDIs can be repaired primarily with an excellent outcome. The reports of double-barreled dual hepaticojejunostomy are scarce. Further study and data accumulation are needed to clarify associated morbidity, mortality, and long-term outcomes.
